# Conversion of I-gel to definitive airway in a cervical immobilized manikin: Aintree intubation catheter vs long endotracheal tube

**DOI:** 10.1186/s12871-020-01069-9

**Published:** 2020-06-18

**Authors:** Yun Jeong Chae, Heirim Lee, Bokyeong Jun, In Kyong Yi

**Affiliations:** 1 Department of Anaesthesiology and Pain Medicine, Ajou University School of Medicine, 164, World cup-ro, Yeongtong-gu, Suwon, 16499 South Korea; 2 Office of Biostatics, Ajou Research Institute for Innovative Medicine, Ajou University Medical Center, 164, World cup-ro, Yeongtong-gu, Suwon, 16499 South Korea

**Keywords:** Airway management, Fiberoptic, Intratracheal intubation, Manikin, Supraglottic airway device

## Abstract

**Background:**

After prehospital insertion of i-gel, a popular supraglottic airway (SGA), fiberoptic-guided intubation through i-gel is often required to switch the i-gel to a definitive airway for anticipated difficult airway. The Aintree intubation catheter (AIC) was developed for this purpose yet it requires many procedural steps during which maintenance of adequate ventilation is difficult. We custom-made a long endotracheal tube (LET) which may facilitate this procedure and compared the efficacy of the AIC and LET in a cervical immobilized manikin.

**Methods:**

In this 2 × 2 crossover manikin-based trial, 20 anaesthesiologists and residents performed both methods in random order. Total intubation time, fiberoptic time, and procedure time were recorded. The ease of insertion, procedure failure rate, difficulty score, and participants’ preference were recorded.

**Results:**

Total intubation time was significantly shorter for the LET than the AIC group (70.8 ± 16.4 s vs 94.0 ± 28.4 s, *P* = 0.001). The procedure time was significantly shorter in the LET group (51.9 ± 13.8 s vs 76.5 ± 25.4 s, *P* <  0.001). The ease of insertion score was lower, i.e., easier, in the AIC than the LET group (2.0 [1.0–2.75] vs 1.0 [1.0–1.0], *P* <  0.001). Fiberoptic time (19.0 ± 6.9 s vs 17.5 ± 12.3 s) and subjective difficulty (4.0 [3.0–6.0] vs 4.0 [3.0–5.75]) were similar between groups. Fourteen participants preferred the LET method (70%) due to its fewer procedural steps.

**Conclusions:**

LET resulted in a shorter intubation time than the AIC during fiberoptic-guided intubation through the i-gel, possibly due to the less procedural steps compared to AIC.

**Trial registration:**

NCT03645174 (ClinicalTrials.gov, Aug 22, 2018).

## Background

Supraglottic airway (SGA) has become a common method of airway management for out-of-hospital cardiac arrest [1–3]. The I-gel (Intersurgical, Berkshire, United Kingdom), a second generation SGA, has shown higher success rates in prehospital setting than first generation SGAs and endotracheal intubation [1, 2, 4]. As utilization of i-gel continues to increase, there is also an increasing clinical need for an easy yet reliable conversion method to a definitive airway after prehospital i-gel insertion. Currently, there are two non-surgical options for the conversion: intubation using a laryngoscopy after pre-existing i-gel removal or intubation through i-gel [5, 6]. If a difficult airway is expected in a well-functioning i-gel, intubation through i-gel is recommended [7] as its removal could make the situation worse.

When using a conventional endotracheal tube with the i-gel during conversion, however, there is a risk of dislodgement of the endotracheal tube due to the different lengths of the endotracheal tube and the i-gel [8]. Devices such as Aintree Intubation Catheter (AIC; Cook Critical Care, Bloomington, IN, USA) were developed to facilitate conversion, but it requires many procedural steps during which maintenance of adequate ventilation is difficult [9].

**Fig. 1:**
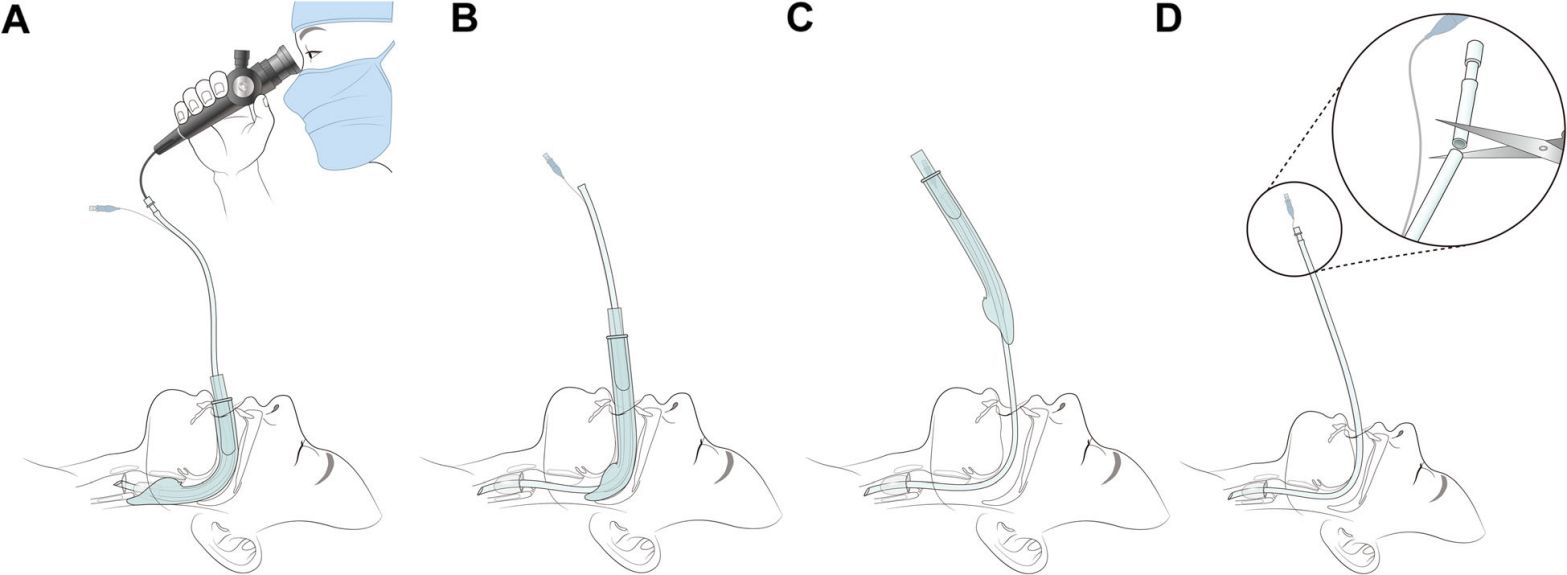
**a** Fiberoptic-guided LET insertion, **(b)** Removal of i-gel, **(c)** Position of cuff pilot balloon during removal of i-gel, **(d)** Adjustment of length of the LET. Abbreviation: LET, long endotracheal tube. Abbreviations: LET, long endotracheal tube

To compensate for the shortcomings, we custom-made a long endotracheal tube (LET) that facilitates removal of a SGA, especially when combined with an i-gel [10–12]. The LET was designed to not only have a longer tube length, but also avoid impingement of the cuff inflation line and cuff pilot balloon during conversion (Fig. 1). The aim of this study was to compare the clinical efficacy of the AIC and LET in the conversion from i-gel to ETT in a cervical immobilized manikin.

## Methods

The study protocol was approved by Ajou Institutional Review Board at Aug, 13, 2018 (AJIRB-MED-OBS-18-226). The current study was also registered in ClinicalTrials.gov (NCT03645174). Participants were anaesthesiologists and anaesthesia residents recruited from our university hospital; the eligibility criterion was not having previous experience of AIC or LET placement. Written informed consent was obtained from all participants.

This study was designed as a 2 × 2 crossover trial, in which each participant performed two types of procedures in a sequence of AB or BA. The first trial was performed in the sequence AB or BA, as allocated by randomization. Previous studies using the AIC reported a mean intubation time of 69.9 s, with a standard deviation of 26.1 s [13]. Therefore, an intubation time difference exceeding 20 s was considered clinically significant; thus, we calculated that at least 16 participants were required (α = 0.05, β = 0.2). Considering potential drop-out rates, 20 participants were recruited.

A two-way analysis of variance was used for data analysis with the AB and BA sequences as the grouping factor and the primary endpoint being the procedural time. Parametric data were analyzed using a paired t-test and nonparametric data, using the Wilcoxon signed-rank test. Categorical variables were analyzed using the McNemar test.

All participants were educated to perform fiberoptic-guided endotracheal intubation through a SGA using two methods by means of an educational video. The rationale behind choosing fiberoptic-guided intubation instead of blind intubation for this study is the overall higher success rate of fiberoptic-guided intubation compared to the blind method in a difficult airway situation (98.6% vs 85.3%) [6]. The first method of intubation involved using the AIC, and the second method involved using the LET developed by our group. Each participant used both methods, in a random order, as determined by a random number table (http://www.random.org).

The manikin (Laerdal Airway Management Trainer; Laerdal Medical, Stavanger, Norway) was fit with a rigid neck collar (Philadelphia, West Deptford, NJ, USA) producing a simulated difficult airway. Prior to the trial, a size 4 i-gel was inserted into the manikin and the position confirmed as a grade 1 laryngeal view using a fiberoptic endoscope. A flexible intubation video-endoscope (KARL STORZ, Tuttlingen, Germany) was used as the fiberoptic endoscope during the procedure.

All participants followed the same intubation protocols. For the AIC method, the catheter was first connected to the fiberoptic device and inserted within the i-gel lumen. The i-gel was removed after confirming the catheter position within the trachea, after which an ETT of ID 7.0 mm (Shiley™ Endotracheal Tubes with TaperGuard™ Cuff; Medtronic, Minneapolis, MN, USA) was inserted using the catheter as a guide. Finally, inflation of the manikin’s lungs was confirmed by artificial manual breathing unit (ambu) bagging. For the LET method, an LET of ID 7.0 mm was fitted to the fiberoptic endoscope and inserted within the i-gel lumen. After confirmation of the LET insertion into the trachea, the fiberoptic endoscope and subsequently the i-gel were removed. Inflation of the manikin’s lungs was confirmed in the same manner as for the AIC method.

The primary performance parameter was the total intubation time, measured by a separate observer using a timer. ‘Total intubation time’ was defined as the time from when the tip of the fiberoptic endoscope was inserted into the i-gel lumen up to inflation of the manikin’s lungs after ambu bagging. ‘Fiberoptic time’ was defined as the time from the entrance of the fiberoptic endoscope into the i-gel lumen to its passage of the vocal cords. ‘Procedural time’ was the difference between the total intubation and fiberoptic times, and reflected the actual time required for each method independent of the participant’s fiberoptic skill level. ‘Intubation failure’ was defined as failure of lung inflation after ambu bagging. This occurred from oesophageal intubation or situations requiring i-gel reinsertion for various reasons, such as dislodgement of the ETT or AIC, or moving of the fiberoptic endoscope away from the vocal cords. The ease of insertion for vocal cord passage of the ETT was scored during each procedure by a separate observer as follows: 1; excellent, no resistance, 2; good, moderate resistance, 3; difficult, remarkable resistance, and 4; insertion impossible in three attempts. After each procedure, the participants rated intubation difficulty using a numeric scale from 1 (extremely easy) to 10 (extremely difficult). Each participant also recorded their preference between the two methods and the reason for their preference [14].

We set the length of LET to 45 cm. The length was based on previous studies and our experiments. Takenaka et al. [11] showed that if removal of SGA is not considered, the optimal length of ETT for adequate endotracheal insertion through SGA was the sum of the length of the SGA, the distance between the SGA mask aperture and the vocal cords, and the distance between the upper border of the ETT cuff and ETT tip. The length thus derived was approximately 33 cm considering the length of SGA being 22 cm [11]. This was similar or somewhat greater than the conventional ETT length. In addition to this length, the proximal end of the ETT above the proximal end of the SGA after endotracheal insertion should be long enough if SGA removal is necessary to prevent dislodgement of the ETT [12]. The distance from the teeth to the proximal end of the ETT should be longer than the length of the SGA, so that during removal of the SGA, the ETT can be caught and prevented from coming out altogether. Assuming that the depth of ETT fixed at the teeth is 23 cm, and the SGA length is 22 cm, at least 45 cm is needed for LET. In addition, the length of the ETT should be shorter than 55 cm so that the LET does not interfere with the distal flexible portion of the fiberoptic endoscope (65 cm long). The cuff inflation line was also kept long enough such that the cuff pilot balloon would not get caught during tube change. Therefore, the range of length for LET was 45–55 cm. Accordingly, we chose the shortest length, 45 cm, to keep the length of the cuff inflation line as short as possible. The branching point of the cuff inflation line from the body of the ETT was the same as that of a conventional tube, so that after the procedure, the device could be cut to a similar length as a conventional ETT without damaging the inflation line (Fig. 1). Both the LET and the Shiley™ Endotracheal Tubes with TaperGuard™ Cuff are made of polyvinyl chloride.

## Results

### Demographics

**Fig. 2:**
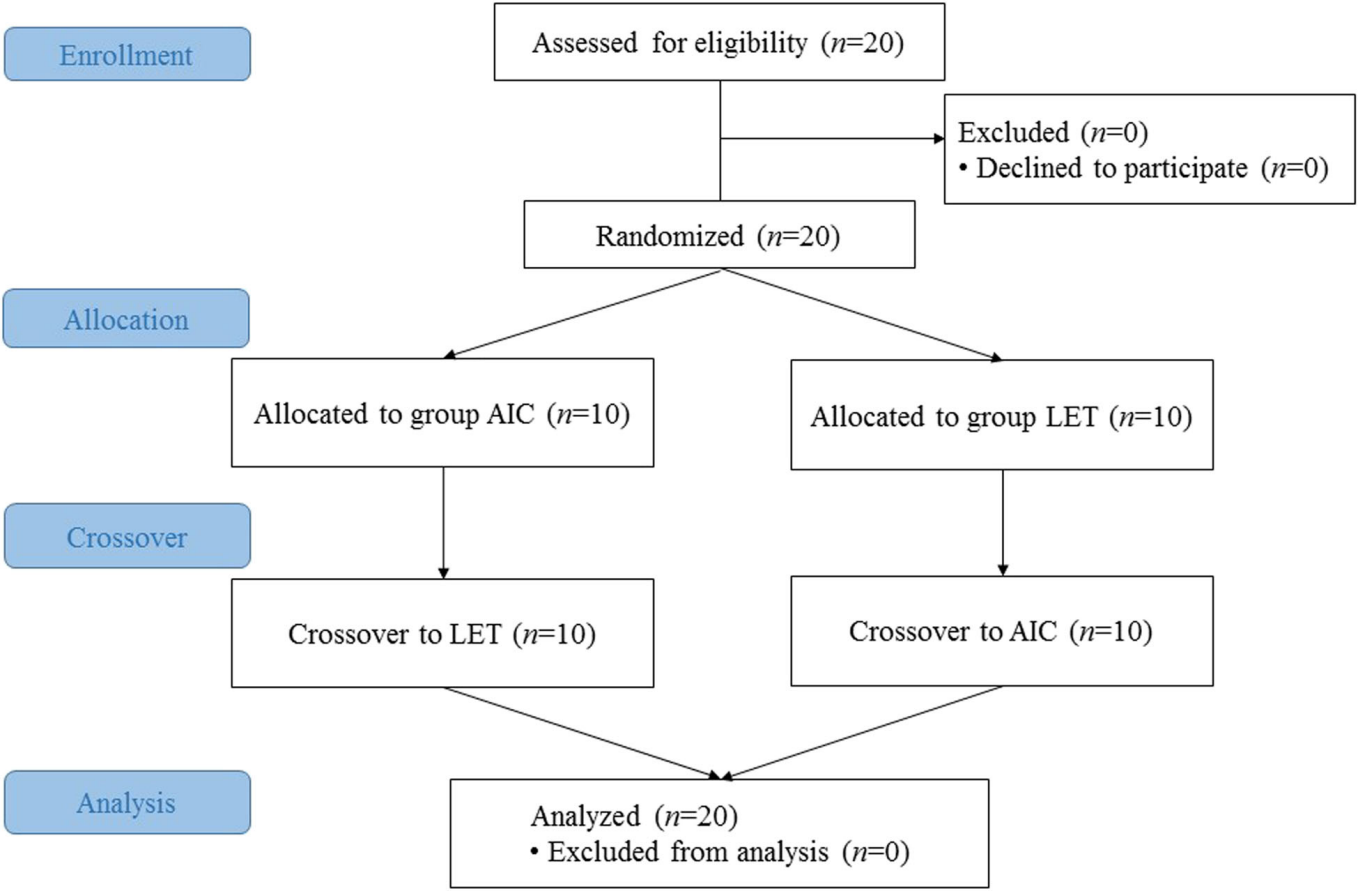
Consort diagram. Abbreviations: AIC, Aintree intubation catheter; LET, long endotracheal tube

Twenty participants were enrolled (Fig. 2), of whom eight were anaesthesia specialists and 12, anaesthesia residents. The mean age of the participants was 32.7 ± 2.6 years. The mean experience with fiberoptic-assisted intubation other than with AIC or LET was 12.9 ± 12.6 times. All the participants completed the study training (Table 1).

**Table 1:** Demographics

No.	Position	Sex	Age	Experiences			
				Anesthesiology (years)	Fiberoptic intubation (times)	AIC (times)	LET (times)
1	Resident	M	30	3	20	0	0
2	Resident	F	35	4	20	0	0
3	Fellow	M	37	5	16	0	0
4	Resident	M	28	1	0	0	0
5	Resident	F	31	1	0	0	0
6	Fellow	M	34	5	20	0	0
7	Resident	M	32	1	0	0	0
8	Resident	F	31	2	10	0	0
9	Resident	F	30	3	15	0	0
10	Resident	F	33	4	20	0	0
11	Resident	F	33	4	10	0	0
12	Resident	F	28	3	15	0	0
13	Fellow	F	32	5	20	0	0
14	Resident	F	32	2	15	0	0
15	Resident	F	32	2	23	0	0
16	Attending	M	36	10	50	0	0
17	Attending	F	35	9	20	0	0
18	Fellow	F	34	5	15	0	0
19	Fellow	M	36	5	3	0	0
20	Fellow	M	35	6	25	0	0

Abbreviations: *AIC* Aintree intubation catheter, *LET* long endotracheal tube

### Primary outcomes of intubation time

**Fig. 3:**
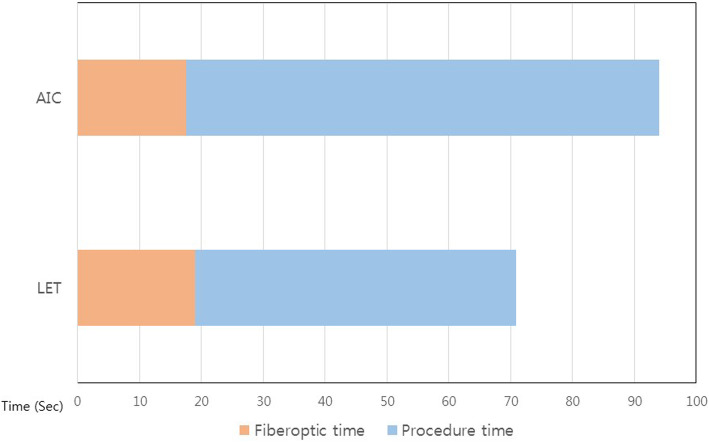
Intubation time. AIC and LET resulted in similar durations for fiberoptic time, however, LET resulted in shorter duration for procedure time. Total intubation time was shorter in LET. Abbreviations: AIC, Aintree intubation catheter; LET, long endotracheal tube.

The total intubation time for the AIC group was 94.0 ± 28.4 s, which was significantly longer than that for the LET group (70.8 ± 16.4 s) (*P* = 0.001). The fiberoptic time was not different between the two groups (AIC: 17.5 ± 12.3 s, LET: 19.0 ± 6.9 s, *p* = 0.61). The procedural time, which was the difference between the total intubation time and fiberoptic time, was significantly longer in the AIC group (76.5 ± 25.4 s) than in the LET group (51.9 ± 13.8 s) (*P* <  0.001) (Fig. 3). One participant failed one time due to dislodgement of the AIC.

### Subjective outcomes

The score of ease of insertion was lower for the AIC (AIC 1.0 [1.0–1.0] vs LET 2.0 [1.0–2.75], *P* <  0.001), but no difference in subjective difficulty was found between the two groups (AIC 4.0 [3.0–5.75] vs LET 4.0 [3.0–6.0], *P* = 1.000) (Table 2). Overall analysis of preference between the two methods showed that six participants preferred the AIC method (30.0%) and 14 preferred the LET method (70.0%). Among those who preferred the AIC method, four described it as having easier vocal cord passage, and two felt that the LET was more difficult to maneuver due to its reduced rigidity. The 14 participants who preferred the LET method described the reason for their preference as the reduced number of procedure steps compared to the AIC method.

**Table 2:** Intubation profile

Parameter	AIC (*n* = 20)	LET (*n* = 20)	*P* value
Total intubation time, sec	94.0 ± 28.4	70.8 ± 16.4	0.001
Fiberoptic time, sec	17.5 ± 12.3	19.0 ± 6.9	0.612
Procedure time, sec	76.5 ± 25.4	51.9 ± 13.8	< 0.001
Success, *n* (%)	19 (95)	20 (100)	1.000
Ease of insertion	1.0 [1.0–1.0]	2.0 [1.0–2.75]	< 0.001
Difficulty	4.0 [3.0–5.75]	4.0 [3.0–6.0]	1.000

Values are mean ± standard deviation or median [interquartile range] or number (%)

Abbreviations: *AIC* Aintree intubation catheter, *LET* long endotracheal tube

## Discussion

In our comparison of using the AIC or LET method for fiberoptic-guided endotracheal intubation through i-gel in a difficult airway manikin, the LET resulted in shorter intubation and procedural times. The score for ease of vocal cord passage of the ETT was higher by approximately 1 point (in a range of 1 to 4 points) compared with that for LET, but there was no difference in the subjective difficulty. Seventy percent of participants preferred the LET method due to its reduced number of procedure steps.

Previous studies using the AIC method report that the intubation time excluding the SGA insertion time was 67–90 s [15, 16]. In our study, the mean intubation time was longer (94.0 s), probably because none of the participants had previous experience with the AIC or the LET. Nevertheless, the LET method had shorter intubation times than the AIC (70.8 s). Even when considering the procedural time, excluding the time required for locating the vocal cords with the fiberoptic device, there was a significant difference between the two methods (AIC: 76.5 s vs LET: 51.9 s). Intubation time and procedure time are of clinical significance since the patients are in apnea condition during the procedure.

When using the AIC, the ETT is inserted after removal of the SGA, and the patient remains in apnea until the ETT is in place. Although connecting the AIC to Rapi-Fit® adaptors (Cook Critical Care, Bloomington, IN, USA) allows jet ventilation for a while, reinsertion of the SGA may eventually be needed. In the LET method, however, ventilation is more secure because the SGA is not removed before ETT placement is confirmed. If procedural problems occur, ventilation can be performed by connecting the ETT to the circuit or through the SGA in situ. This ensures safety against apnea in case of prolonged procedures. Hence, the LET has clinical value in that it decreases the intubation time and provides a ventilation tool at hand. Another significant finding was the high user-preference for the LET due to the fewer procedural steps. Overall, the LET method showed advantages over the AIC method in terms of decreased intubation time, simplicity of the procedure, and safety.

A small number of studies have utilised a similar concept during intubation through SGA. Knoshita et al. [10] reported using a longer tube to facilitate conversion of an LMA Fastrach™ (Laryngeal Mask Company, Henley-on-Thames, UK) but did not compare it to other methods. Similar to our concept, Weiss et al. [17] reported on a method in paediatric patients that used two separate uncuffed ETTs connected to each other. They reported that ventilation was possible during LMA removal; hence, there was no need to rush the procedure [17]. This is not only related to the psychological stability of the intubator but is also clinically significant because most paediatric patients intubated through SGAs have difficult airway situations. Unfortunately, this cannot be applied to adults as they require the use of cuffed ETTs. When using the Weiss method, the cuff pilot balloon is impinged due to the lack of space within the SGA lumen, resulting in an obstacle during SGA removal and possible damage to the balloon. The LET used for this study has several advantages in terms of tube design. The cuff inflation line is longer than the total length of the tube, so that the cuff pilot balloon is positioned distal to the tube end and does not interfere with SGA removal. Balloon inflation can be performed before SGA removal, if required.

Direct insertion of ETTs into the SGA has been studied, such as with the use of an intubating laryngeal mask airway (iLMA). The iLMA has a stiff angled shank and a wide internal diameter for ETT insertion, as does the LMA Fastrach™. It is also shorter than other LMAs and has a stabiliser rod that pushes the ETT [14]. A previous study compared the efficacy of the iLMA with that of a combination of the AIC and classic LMA (cLMA) during insertion of a 7.0 mm ID ETT. They reported that not only was iLMA insertion more difficult than cLMA, but it also resulted in a poor glottis view in 26% of cases, even after insertion [14]. Furthermore, the iLMA is less widely available than the cLMA [9] and has a steeper learning curve, hampering its routine use [18] Thus, the iLMA does not appear to improve upon the combination of the AIC and cLMA [14, 19]. The i-gel and LET combination used in this study shares conceptual similarities with the iLMA, but compensates for iLMA shortcomings. Several studies have shown that i-gel yields a higher insertion success rate, the best fiberoptic view, and superior results in intubation through SGAs than those with other SGAs [16, 20, 21]. The i-gel and LET combination used in this study shares conceptual similarities with the iLMA, but compensates for iLMA shortcomings. The i-gel and LET combination is therefore a potentially better option than iLMA, but further study is definitely warranted.

In the AIC method, resistance during vocal cord passing (“railroading”) was reported in 24% of cases [15]. A previous study utilising iLMA and a 7.0-mm ID ETT as a fiberoptic guide, without the AIC, reported intubation failure in three of eight cases due to railroading [19]. This problem occurs due to the difference in diameter between the outer and inner layers. In this case, the difference is between the outer diameter of the AIC and internal diameter of the ETT. In the LET method, the difference in diameter is larger than in the AIC method; therefore, impediment at the glottis may be greater than that with the AIC [19, 22]. In our study, we described this phenomenon as ‘ease of insertion’. We found that both methods had acceptable ease of insertion scores; however, the LET group showed slightly more resistance than the AIC group, without any difference in the subjective difficulty. Three cases showed marked resistance (grade 3) during LET insertion, but railroading was finally possible in all cases in our study, possibly because it was performed within the established SGA path. A different tube tip design [23] or a thicker fiberoptic device may be useful to facilitate railroading. Nevertheless, further investigations are required.

This study has some limitations. It was performed at a single center, used a manikin, and only included situations with proper positioning of the SGA and good laryngeal view by the fiberoptic. Additional clinical trials are warranted, including situations with a poor laryngeal view through the SGA. Furthermore, combinations of the LET with other commercially available SGAs should be compared. Finally, further studies and possible design modifications are required to test the incorporation of blind method intubation through SGA.

## Conclusions

The LET designed by our group resulted in a shorter intubation time for residents and anaesthesiologists than did the AIC during fiberoptic-guided intubation through an i-gel. This is possibly due to its more concise procedural steps. The LET appears to be a useful tool during exchange from i-gel to definitive airway in anticipated difficult airway situations.

## Data Availability

The datasets used and/or analyzed during the current study are available from the corresponding author on reasonable request.

## Abbreviations

AIC: Aintree intubation catheter
LET: Long endotracheal tube
LMA: Laryngeal mask airway
SGA: Supraglottic airway
